# Development of a high number, high coverage dog rabies vaccination programme in Sri Lanka

**DOI:** 10.1186/s12879-019-4585-z

**Published:** 2019-11-20

**Authors:** Carlos Sánchez-Soriano, Andrew D. Gibson, Luke Gamble, Jordana L. Burdon Bailey, Samantha Green, Mark Green, Barend M. deC. Bronsvoort, Ian G. Handel, Richard J. Mellanby, Stella Mazeri

**Affiliations:** 1 The Royal (Dick) School of Veterinary Studies, The University of Edinburgh, Easter Bush Veterinary Centre, Roslin, Midlothian EH25 9RG UK; 2 The Roslin Institute, Division of Genetics and Genomics, Easter Bush Veterinary Centre, Roslin, Midlothian UK; 3 Mission Rabies, Cranborne, Dorset UK; 4 Dogstar Foundation, Negombo, Western Province Sri Lanka

**Keywords:** Rabies, Dogs, Sri Lanka, Vaccination, Mobile phone application, Coverage

## Abstract

**Background:**

Rabies is estimated to cause 59,000 deaths and economic losses of US$8.6 billion every year. Despite several years of rabies surveillance and awareness programmes, increased availability of post-exposure prophylaxis vaccinations and dog population control, the disease still remains prevalent in Sri Lanka. This study reports the roll-out of a high number, high coverage canine rabies vaccination campaign in Sri Lanka, providing estimates for the vaccination coverage achieved, analysing the local dog demographics, and identifying barriers of attendance to static vaccination clinics.

**Methods:**

A mass dog vaccination campaign was undertaken in Negombo, Sri Lanka. The campaign was composed of static point and door-to-door vaccination stages, with a final survey of vaccination coverage. A large volume of data on the distribution, health, and signalment of vaccinated dogs was collected through a mobile phone application. A logistic regression model was developed to investigate which socio-spatial and dog-related factors influenced attendance of owners to static vaccination points.

**Results:**

The campaign vaccinated over 7800 dogs achieving a vaccination coverage of 75.8%. A dog:human ratio of 1:17 was estimated. Most dogs were owned, and the dog population was mostly male, adult, and non-sterilized. Unawareness, unavailability and handling problems were the most common reasons given by owners to explain failure to attend a static vaccination point. The regression analysis showed that increasing distance to a static point, in addition to young age and poor health of the dog, were associated with a decrease in the likelihood of attendance to a static vaccination points.

**Conclusion:**

This study demonstrates the feasibility of high number, high coverage vaccination campaigns in Sri Lanka. The information on dog ecology and barriers of attendance to static point vaccination clinics will facilitate development of future vaccination campaigns.

## Background

Rabies is still a prevalent and underreported disease in many developing countries, causing 59,000 deaths each year, and economic losses amounting to 8.6 billion USD annually [1]. Ninety-nine percent of human rabies cases can be traced back to bites from rabies infected dogs [2]. Infected patients develop fatal encephalitis unless they are treated shortly after the bite with post-exposure prophylaxis (PEP), a treatment not widely available in many rabies-endemic regions [3, 4]. Since dog bites are the main source of human infections [2], mass dog vaccination campaigns represent the most effective course of action to reduce rabies incidence [1, 5], and have been shown to reduce human infection rates in multiple settings [6–8]. The World Health Organization recommends a minimum annual vaccination coverage of 70% of the dog population [9, 10] in order to achieve herd immunity, reduce rabies’ incidence, and minimize the burden of the disease in both dog and human populations [5].

In order to achieve this goal, different dog vaccination strategies are used depending on the local dog ecology, dog ownership structure, and the resources available. Static point (SP) vaccination approaches are commonly used, as they are easy to establish and are an efficient way to vaccinate a large number of dogs with limited personnel. However, reliance on SP clinics only often leads to a failure to vaccinate a sufficient proportion of the population, since they depend on high ownership levels and other socio-economic and cultural factors which may influence dog owners’ attendance [11]. Although more logistically challenging and costly, “door-to-door” (D2D) approaches based on visiting all households in a community and vaccinating free-roaming and owned dogs are able to achieve high coverages very effectively [12, 13]. For this reason, many vaccination campaigns combine SP and D2D stages to maximise vaccination coverage in a feasible and cost-effective manner [14, 15].

Rabies-related human deaths in Sri Lanka have steadily diminished since the establishment of the rabies control program in 1975. Despite more than 30 years of efforts, the disease still represents a serious concern for the island, especially in regions with economies largely sustained by tourism. The latest surveys in 2014 estimated a dog vaccination coverage of 48% [16], which is considered to be too low to rapidly eliminate the disease. Rabies remains prevalent among the Sri Lankan canine population, resulting in around thirty human deaths each year [16]. Consequently, PEP expenditure represents an important economic burden, amounting to more than 300,000 prescriptions annually [16, 17]. Nationwide efforts to reduce the prevalence of the disease continue to be based on treatment availability, dog vaccination, and stray dog population control [16–18]. However, dog vaccination and neutering methods in Sri Lanka have been poorly reported, with scarce data available on dog demographics and vaccination coverage. The lack of a working template for high numbers, high coverage campaigns is one of the factors hindering the development of efficient vaccination operations that could be applied in many of regions of Sri Lanka to enable the nation to reach sufficient dog vaccination coverage. This report describes a mass dog vaccination campaign carried out in Negombo by Mission Rabies [19] and the Dogstar Foundation [20], which was able to vaccinate a large number of dogs, obtaining a high vaccination coverage, and whose design could be implemented in other regions of the country to reduce the incidence of rabies. The objectives of this study included: the estimation of the vaccination coverage achieved, the analysis of the demographics of the local dog population, and the identification of barriers of attendance to SP.

## Methods

### Study area

Negombo is the biggest city in the Gampaha District, located in the western coast of Sri Lanka. Negombo also constitutes one of the biggest hubs of the country, with a population of 142,136 inhabitants [21].

### Mission rabies 2016 vaccination campaign: period and course of the campaign

**Fig. 1:**
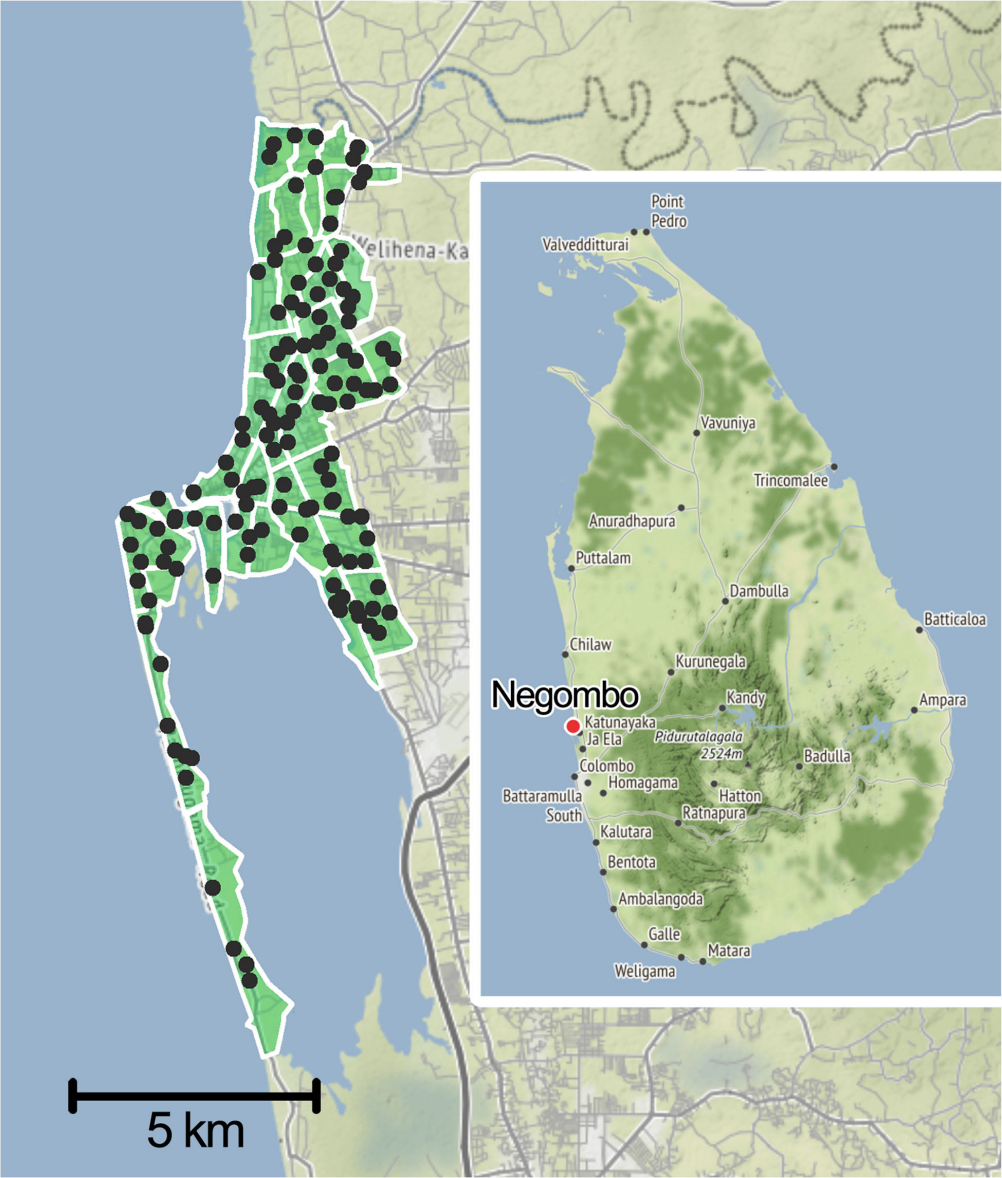
Topographical division of Negombo in 33 wards. The division into wards was performed according to the working zone shapefile provided by Mission Rabies. The location of the 146 static vaccination points is marked as dots. The location of Negombo within Sri Lanka is shown on the right. Background map tiles by Stamen Design, under CC BY 3.0. Data by OpenStreetMap, under ODbL

A pilot D2D campaign in 2015 was used to demonstrate the feasibility and effectiveness of working protocols. The 2016 campaign was performed between June and September, and covered most of the city, whose area was divided into 33 wards (Fig. 1), denominated with numbers 1 to 34 and missing number 21. Based on previous information regarding ownership levels from the 2015 pilot campaign [19] and reports from another municipality in Gampaha District [22], a SP stage was included in the campaign. A total of 146 static vaccination points were set up daily in different areas of the city (Fig. 1), from June 15th to September 1st, giving coloured collars to the animals administered vaccination (Nobivac® Rabies, MSD Animal Health). The SP efforts were complemented by a D2D stage, from September 12th to 28th, in which the staff members covered the entire area of each working zone, vaccinating and marking (with paint and/or a collar) any dogs found on the street and knocking on household doors to offer vaccination to owned dogs. Free-roaming dogs which did not approach the teams willingly were caught using lightweight Balinese-style nets, and restrained securely. All staff members involved in the vaccination process were trained to do so causing the minimum distress to the animals. A survey to assess the vaccination coverage was performed from September 13th to 29th, recording the presence or absence of paint marks and collars on any dogs seen while travelling on every traversable road of each ward using a *tuk tuk*. Surveys were usually carried the day after the D2D vaccination, otherwise surveys were carried out maximum 2 days after. In 2 wards where the coverage results were below 70%, the vaccination teams were sent for a second round of D2D vaccination and subsequent survey. Survey entries gathered in wards 13, 24, 25, 26, 27, 28, 29, 30 and 34 were collected by a different surveyor, since the original surveying supervisor was not available. Due to the lack of training, entries from these 9 wards were not included in the coverage assessment. Field data during all stages was collected using the Mission Rabies App [23], a web-based platform created for simple management and streamlined entry of field data. The app collected global positioning system (GPS) coordinates and timestamps for each dog vaccinated automatically and offered a path-tracking tool allowing the staff to check their spatial coverage in real time. The app also requested additional relevant data of the dog such as sex, age, health status or neuter status in addition to other information such as the team responsible for the vaccination, the provider of any previous vaccine, and the opinion of owners on matters such as neutering procedures and the reason why they did not bring their dog to a SP.

### Data sources

#### Collection tools and data collected

Field data collected using the Mission Rabies App [23] was compiled into datasets and used for this study. A shapefile containing the specifics of the polygons used by Mission Rabies to divide Negombo in the 33 wards was also provided.

#### Other sources

Data regarding additional geospatial variables for the regression analysis was obtained from publicly available sources. Weather data for Negombo during the campaign was obtained from the World Weather Online database [24], averaging the tri-hourly measurements for temperature and precipitation during the working hours (6:00–15:00) for each day of the SP campaign. A raster file depicting population density in 2015 (100 m resolution) was obtained from WorldPop [25] and used to extract population density at each D2D entry coordinates. Since the raster did not cover the entirety of the working area, entries located outside of the raster’s data grid were applied a buffer averaging the values found in a 550 m radius. In the absence of suitable poverty geodata for Negombo, a raster file depicting the number of underweight children under five years old (in the year 2000) with a 2.5-min resolution was obtained from the Socioeconomic Data and Applications Center (SEDAC) [26]. Hunger data for each D2D entry coordinate was extracted from the raster and used as proxy data for poverty. A shapefile containing land types (in 2012) based on the national 7-class classification scheme was obtained from the World Bank Group [27] and used to determine land type for every D2D entry coordinate. The R package *ggmap* [28] was used to plot all maps, using background tiling sourced from Stamen Design (using data by OpenStreetMap [29]), available under CC BY 3.0 license.

### Data analysis

Data manipulation and analysis was performed using the R statistical software environment version 3.4.3 [30].. The use of specific R packages for particular purposes is detailed below:

#### Identification and management of GPS outliers

The GPS data was checked to detect any erroneous entries caused by coordinate inaccuracy by automated recording systems [31]. These inaccuracies were mostly represented by coordinates ending up outside the visited ward, or in the ocean. These were considered to occur at random, and a system for outlier coordinate detection was created. This system was irrelevant for the SP stage since coordinates for all the vaccination points were known. For the D2D locations, due to the lack of reference coordinates, this discrimination was performed by spatial clustering. Using the *dbscan* function from the *fpc* package [32], clusters of vaccination coordinates were determined. The function required 2 parameters to be specified in advance: the size of the epsilon neighbourhood was set at 0.005, and the minimum number of neighbours was set to 3. Clusters containing < 20% of the total points for a given day and team responsible were considered outliers. The detection system was complemented by a visual screening of the coordinates. At the end of the process, 836 entries were marked as outliers and not included in the regression analysis due to the need for spatial accuracy, however they were included in the vaccination coverage analysis after adjusting their ward according to Mission Rabies working schedules. The assumption that the outliers occurred at random was checked by comparing the distribution of the variables included in the regression model between the outlier non-outlier entries.

#### Estimation of coverage by ward

Vaccination coverages for each ward were calculated based on the number of dogs marked with a collar or paint sighted during the survey out of the total number of dogs sighted. The 95% binomial confidence interval (CI) was calculated using the *binom.test* function from base R [30], which carries out an exact binomial test. The *over* function from the *rgeos* package [33] was used to match the coordinates from the data with the ward shapefile, which was imported using the *rgdal* package [34].

#### Analysis of dog demographics

The dog population in Negombo was estimated using the Chapman estimator [35] for mark and recapture. The dog population density was calculated using the area for the Mission Rabies working zone (30 km^2^). The 95% CI for the population size was calculated using the *ciChapman* function from the *recapr* package [36], with the default bootstrap method. Data obtained from the D2D stage was used to study the dog demographics on sex, age, ownership and neuter status of the dogs, as it contained information on both the owned and stray populations. In order to determine any relationship between the dog-related variables (sex, age, ownership status, vaccination status and neuter status), the Chi-Squared Test for Independence was used, with a significance level (α) of 0.05. In cases where any of the expected frequencies were lower than 5, the Fisher’s Exact Test was used instead.

#### Analysis of owner opinions on neutering and failure to attend SP

The answers given by the owners when asked “are you opposed to dog sterilization, and if so why?” and “why didn’t you attend to the SP campaign?” were compiled into a frequency table. Infrequent answers were included under the “Other” category.

#### Development of the logistic regression model

A logistic regression model analysing the effect of several dog-related and geospatial factors on attendance of owners to SP was built using the *glm* (generalised linear model) function. Attendance of a dog to SP was defined as the outcome variable, supplied as TRUE/FALSE. For the model, only entries regarding owned dogs found during the D2D stage were considered (4310 entries), filtering out any coordinate outliers (675 entries). The *RANN* package [37] was used to determine the closest static vaccination point from each owner’s household. Packages *rgdal* [34] and *raster* [38] were used to manipulate shapefiles and raster files, respectively. The linearity of continuous predictive variables was tested using the Box-Tidwell Transformation test [39], and variables “distance to SP” and “precipitation” were transformed into categorical variables as linearity could not be assumed. “Distance to SP” was divided into four quantiles: [2.1–126], (126–194], (194–279] and (279–1100] (distance in metres), whereas the “precipitation” variable was categorized into slight (< 0.5 mm/h), moderate (0.5–4 mm/h), and heavy (> 4 mm/h) precipitation, according to the UK meteorological office [40]. In order to increase the counts of young animals, “juvenile” and “puppy” dogs were merged into “young”, as opposed to “adult” dogs (over one year of age).

#### Best model selection

A series of different models were built by a mixture of forward and backwards selection using combinations of the relevant explanatory variables and their interactions. After filtering unowned dogs and outlier entries, the dataset was composed of 3635 entries. To determine the performance of the proposed regression models, this data was randomly partitioned into “training” and “testing” subsets, in a 7:3 ratio, using the *vtreat* package [41]. This partition allowed to generate the model from the “training” subset, and test its predictive power on the “testing” subset using the *caret* package [42]. To aid the variable selection process, 5-fold cross validation was used [43], partitioning the “training” data into 5 sets. This process produced goodness of fit estimations (area under the receiving operator curve (AUC)) averaged from the 5 sequential analyses, that allowed for the comparison of the different models in order to select the best. This decision was taken based on two parameters: the AUC, and the Akaike information criterion (AIC) [44]. Once the best model was chosen, its predictive power was determined using the initial “testing” subset by estimating the AUC using the package *ROCR* [45]. Using the final model, ward and vaccination team were included as random effects through mixed-effects regression. Since their influence on the odds ratios was negligible and for the sake of simplicity, the fixed effects model was chosen.

## Results

### Number of dogs vaccinated

During the campaign, a total of 7804 dogs were vaccinated. 4382 dogs were vaccinated at the 146 different SP across the city. During the D2D vaccination stage, a further 3422 dogs were vaccinated. 115 dogs (3.2% of all encountered) were not vaccinated during the D2D stage, the most common reasons being lack of owner consent (61%) and handling difficulties (22.9%). Out of the 5177 dogs seen during the D2D campaign, 1019 (19.7%) had been vaccinated during the SP campaign.

### Estimation of the vaccination coverage

**Fig. 2:**
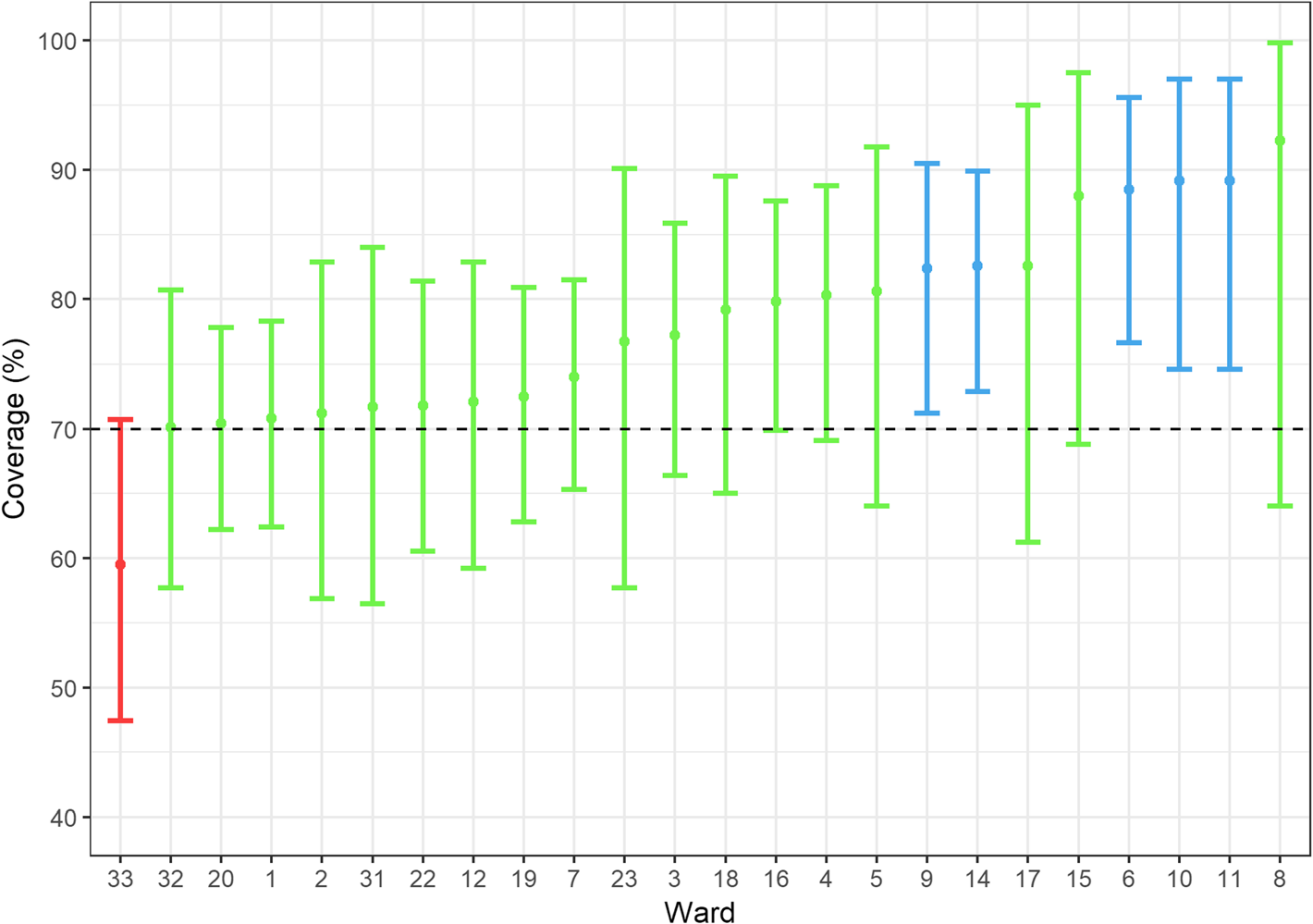
Plot of vaccination coverage achieved in each ward. The 95% Confidence Interval (CI) is represented as vertical bars. Wards in which the coverage surpassed 70% are coloured in green, while wards under 70% are coloured in red. Wards whose coverage and 95% CI lower bound surpass 70% are coloured in blue

The average vaccination coverage across the 24 accurately surveyed wards was estimated as 75.8% (95% CI: 73.6–77.9%), with 1194 dogs out of the 1576 sighted being marked as vaccinated. Vaccination coverages above 70% were estimated in 23 out of the 24 wards (Fig. 2). Table 1 includes a summary of the vaccination efforts during the SP and D2D stages, including vaccination coverages per ward. Dogs vaccinated at SP represented 62.1% of the total dogs found to be previously vaccinated. The remaining had been vaccinated by a private veterinarian (25.4%), in a Dogstar clinic (5.4%) or by another source (7%).

**Table 1:** Summary of vaccination numbers and estimated coverage per ward

Ward	D2D vaccinated	SP vaccinated	Total vaccinated	Marked	Unmarked	Total sighted	Coverage (%)	Confidence Interval (95%)
1	181	159	340	97	40	137	70.8	(62.4–78.3)
2	68	65	133	37	15	52	71.2	(56.9–82.9)
3	155	26	181	61	18	79	77.2	(66.4–85.9)
4	71	103	174	57	14	71	80.3	(69.1–88.8)
5	86	32	118	29	7	36	80.6	(64–91.8)
6	57	36	93	46	6	52	88.5	(76.6–95.6)
7	210	81	291	91	32	123	74	(65.3–81.5)
8	116	31	147	12	1	13	92.3	(64–99.8)
9	89	201	290	56	12	68	82.4	(71.2–90.5)
10	91	162	253	33	4	37	89.2	(74.6–97)
11	83	72	155	33	4	37	89.2	(74.6–97)
12	102	195	297	44	17	61	72.1	(59.2–82.9)
13	36	37	73	NA	NA	NA	NA	NA
14	208	384	592	71	15	86	82.6	(72.9–89.9)
15	42	62	104	22	3	25	88	(68.8–97.5)
16	91	70	161	71	18	89	79.8	(69.9–87.6)
17	137	159	296	19	4	23	82.6	(61.2–95)
18	54	122	176	38	10	48	79.2	(65–89.5)
19	182	239	421	74	28	102	72.5	(62.8–80.9)
20	180	287	467	100	42	142	70.4	(62.2–77.8)
22	99	69	168	56	22	78	71.8	(60.5–81.4)
23	112	213	325	23	7	30	76.7	(57.7–90.1)
24	105	151	256	NA	NA	NA	NA	NA
25	32	0	32	NA	NA	NA	NA	NA
26	47	119	166	NA	NA	NA	NA	NA
27	77	15	92	NA	NA	NA	NA	NA
28	69	119	188	NA	NA	NA	NA	NA
29	47	41	88	NA	NA	NA	NA	NA
30	42	117	159	NA	NA	NA	NA	NA
31	80	103	183	33	13	46	71.7	(56.5–84)
32	71	259	330	47	20	67	70.1	(57.7–80.7)
33	234	408	642	44	30	74	59.5	(47.4–70.7)
34	168	245	413	NA	NA	NA	NA	NA
Total	3422	4382	7804	1194	382	1576	75.8	(73.6–77.9)

The 95% Confidence Interval for the coverage is also included. Vaccination coverage data for wards 13, 24, 25, 26, 27, 28, 29, 30 and 34 was not calculated due to an unreliable method of survey. Ward 21 does not exist

### General dog demographics

**Fig. 3:**
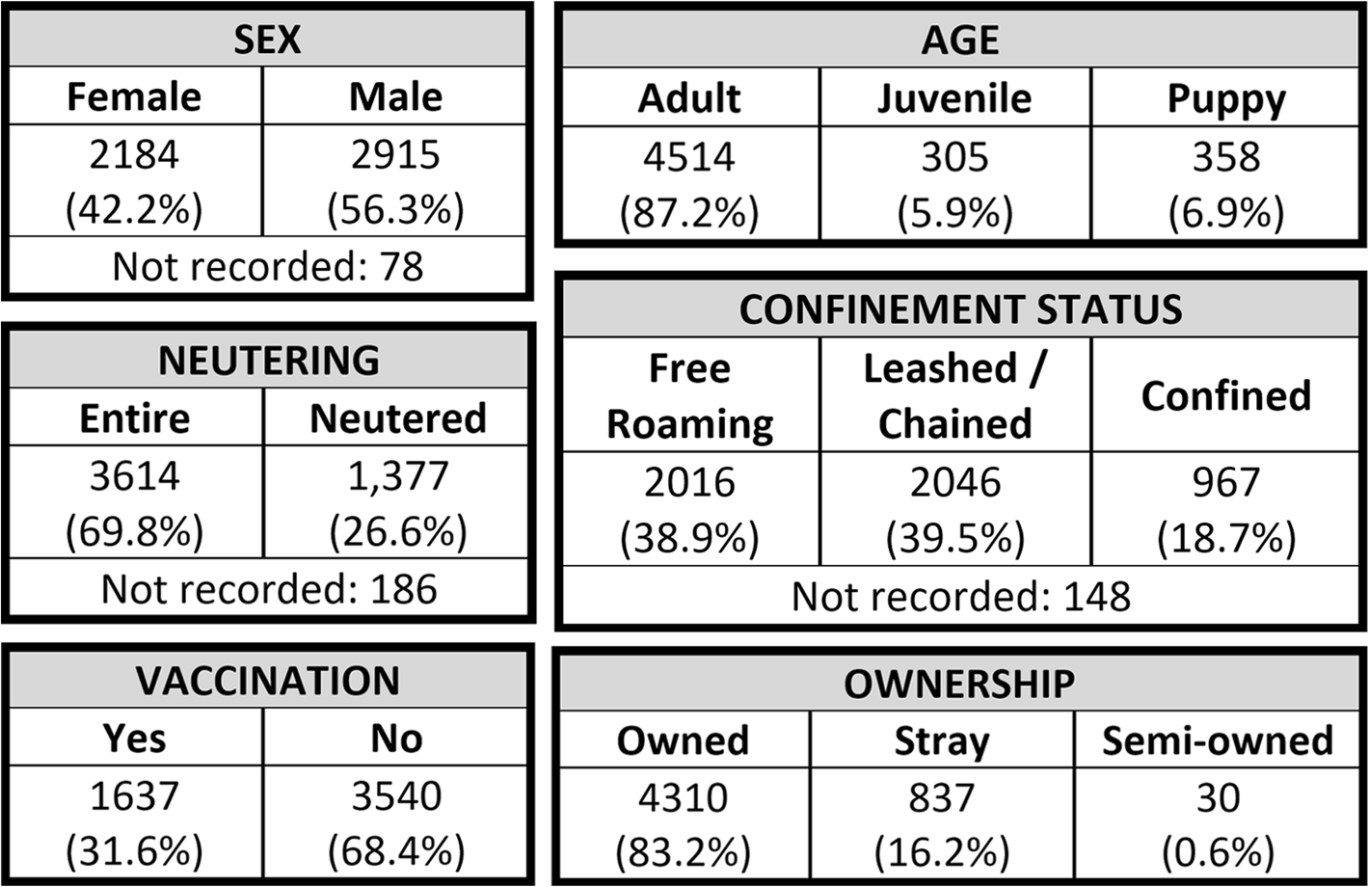
Descriptive tables on dog demographics. The tables show the counts and proportions of dogs in each category, including the number of dogs whose category was not recorded

Using the Chapman estimation, a dog population of 8370 was estimated (95% CI: 8138 – 8608). The dog population density was estimated to be 279 dogs/km^2^ in Negombo. Based on a human population density of 4737 inhabitants/km^2^ [21], these estimates produced a dog:human ratio of 1:17. There was a modestly lower proportion of female dogs (42.2%) compared to males (56.3%). Most of the dogs found during the D2D campaign were considered adults (over one year of age) (87.2%), with only a small proportion classified as juvenile (3 to 12 months old) (5.9%) or puppies (less than 3 months old) (6.9%). A positive relationship was found between female sex and young age (*p* < 0.001). The great majority of the dogs (83.2%) were recorded as owned. More female dogs were considered stray, compared to male dogs (*p* < 0.001). A higher proportion of young (under 1 year of age) stray animals was also found, (*p* < 0.001) compared to a larger, adult-owned population. Almost a third (31.6%) of the dogs seen had been previously vaccinated (at the SP stage, in Dogstar clinics, or by other veterinarians), while the rest (68.3%) were unvaccinated. Young animals were seldom vaccinated (*p* < 0.001), and male dogs were more likely to be already vaccinated (*p* < 0.001). In regards to the confinement status, 37% of the dogs were found free-roaming, 39.5% were found chained or leashed to a wall or post, while 18.7% were confined in a kennel or inside a household. More details on dog population based on sex, age, ownership, vaccination, neutering, and confinement can be found in Fig. 3. Proportion tables for all the relationships drawn in this section are provided in Additional file 1: Figure S1.

From all the dogs brought to SP, 1961 (44.8%) had never been vaccinated before or their vaccine was overdue. 2421 (55.2%) were found to be already vaccinated, having been immunized at a Dogstar clinic (251, 10.4%), by a municipal veterinarian (2059, 85.6%), or by a private veterinarian (89, 3.7%).

### Analysis of dog neuter status

Only a minority (1377, 26.6%) of the dogs encountered during the D2D campaign were neutered. The neuter status of 186 dogs was not recorded. A negative relationship between ownership and neutering (*p* 0.001) was detected, as dogs owned were typically entire. Female animals were more likely neutered (*p* < 0.001), since male dogs were much more frequently owned and entire. Young animals were seldom found neutered (*p* < 0.001). Entire dogs represented a 71.5% of the owned population. When owners were asked whether they were interested in getting their pet sterilised, 23.7% stated that they would like to have their dog neutered, while 69.4% reported that they did not want their dog to be neutered. Table 2 shows their responses when asked why they opposed neutering. Considering neutering as an unnecessary procedure was the most common answer (42.6%), followed by a desire to breed the dog (13.4%).

**Table 2:** Reasons given by dog owners against the sterilization of their dogs

Reason	Frequency	Proportion (%)
The procedure is unnecessary	1314	42.6
No answer	1002	32.5
I plan on breeding the dog	413	13.4
The procedure is sinful	160	5.2
It will change the dog’s behaviour	141	4.6
The dog should be able to mate	22	0.7
The procedure is inconvenient / a time loss	16	0.5

### Identification of barriers of attendance to SP

**Fig. 4:**
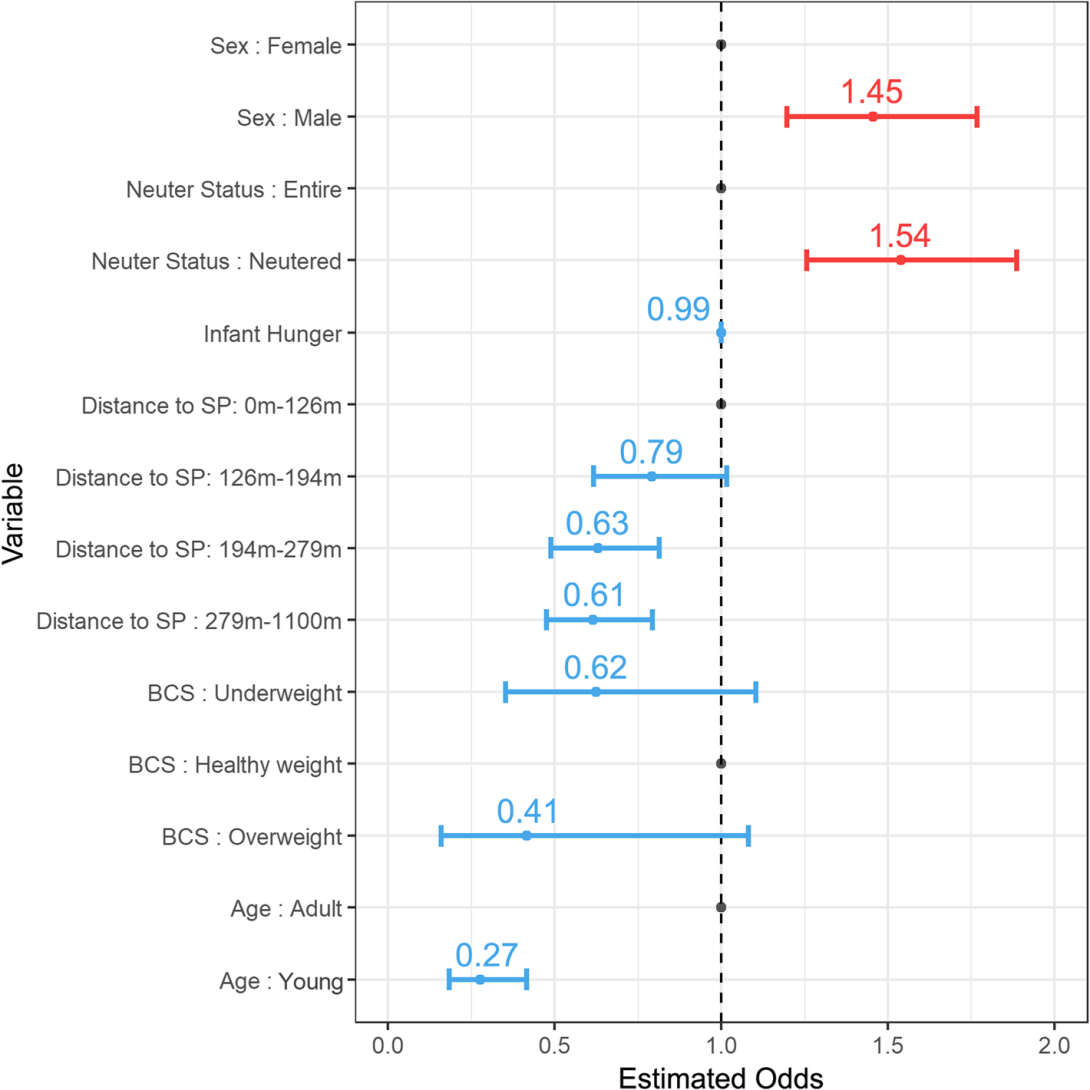
Graphical representation of the chosen regression model. The 95% Confidence Interval (CI) is represented as horizontal bars. The value for the odds ratio is indicated above the 95% CI. A positive relationship between the variable and the response (attendance) is coloured in red (odds ratio > 1), while a negative relationship is coloured in blue (odds ratio < 1). Baseline categories included

The barriers of attendance to SP were investigated by a regression analysis. The results from univariable logistic regression analysis performed to assess each of the variables considered can be found in Additional file 2: Figure S2. The final model represented a compromise between predictive power (greater AUC), and simplicity (less predictors). This model showed that the odds of attendance to SP decrease with increasing distance from household to SP, in young dogs, and in dogs with poor body condition (under/overweight). Odds of attendance were higher in male and/or neutered dogs. Infant hunger showed a very slight negative association with attendance to SP, although not statistically significant (*p* 0.065). The odd ratios for predictors related to unknown categories (unknown sex and unknown age) were not interpreted due to their ambiguous and non-defined nature. This analysis is represented in Fig. 4, showing the odds ratio and CI for each of the predictive variables. A complete table including this information in a numerical format and the *P* values for each predictor can be found in Table 3. The model presented a moderate predictive power (AUC = 0.642).

**Table 3:** Numerical results of the regression model

Variable	Odds Ratio	Confidence Interval (95%)	*P* value (α = 0.05)
Sex: Female	1	Baseline category	
Sex: Male	1.455	(1.2–1.77)	< 0.001
Neuter Status: Entire	1	Baseline category	
Neuter Status: Neutered	1.54	(1.256–1.886)	< 0.001
Infant Hunger	0.999	(0.999–1)	0.065
Distance to SP: [2.1 m, 126 m]	1	Baseline category	
Distance to SP: (126 m, 194 m]	0.792	(0.616–1.015)	0.066
Distance to SP: (194 m, 279 m]	0.63	(0.488–0.813)	< 0.001
Distance to SP: (279 m, 1100 m]	0.614	(0.475–0.792)	< 0.001
BCS: Underweight	0.623	(0.352–1.03)	0.085
BCS: Healthy	1	Baseline category	
BCS: Overweight	0.415	(0.16–1.08)	0.071
Age: Adult	1	Baseline category	
Age: Young	0.275	(0.183–0.415)	0.017

Baseline categories are used as criterion values to calculate the odds ratios

Table 4 shows the most common responses when owners of unvaccinated dogs were asked why they did not take their dogs to a SP. Most common reasons reported were unawareness (37.9%), unavailability (29.3%), and problems handling the dog (10.2%). The dog being too young (5.3%) and excessive distance to the SP (4.5%) were also responses commonly given. Responses regarding rabies vaccination as unnecessary or harmful were uncommon.

**Table 4:** Reasons given by dog owners explaining failure to attend to a Static Point

Reason	Frequency	Proportion (%)
I was unaware of the campaign	1035	37.9
I was unavailable	800	29.3
I couldn’t handle the dog	279	10.2
The dog is actually stray/semi-owned	146	5.3
The dog is too young for vaccination	144	5.3
The distance was too great	122	4.5
No answer	78	2.9
Other	51	1.9
The procedure is unnecessary	45	1.6
The procedure is harmful	13	0.5
The dog was indeed previously vaccinated	13	0.5
The dog is under the care of a private vet	5	0.2

## Discussion

The vaccination effort described in this report demonstrates the feasibility of mass dog vaccination campaigns with high coverage in Sri Lanka. A total of 7804 dogs were vaccinated during the course of the campaign, 3422 at SP locations and 4382 during the D2D campaign, over the 33 wards of Negombo.

The coverage obtained for the Negombo working area achieved the 70% minimum coverage recommended by the WHO [9], and surpassed the 48% vaccination coverage estimated for all of Sri Lanka in previous campaigns [16]. However, reliable survey data is lacking for 9 of the wards. In future campaigns, this can be improved by increasing the number of people trained to assess vaccination coverage in case of unavailability of the primary supervisor. Vaccination coverage over 70% was achieved in one out of the two wards where the initial survey was not satisfactory, after returning to both for a second round of vaccination and survey. The information gathered during the campaign has provided extensive insights on the dog population of Negombo.

The estimated dog:human ratio from Negombo (1:17) is much lower than the ratio reported in Mirigama in 2000 (1:4.6) [22], the ratio estimated by Knobel et al. in 2005 for urban settings in the Asian continent (1:7.5) [46], and the nation-wide estimates of 2014 (1:6.7) [16]. This disparity could be due to the large timespan between the studies and this report, the different human population densities, and the recent increase of population control efforts, provided by organizations such as the Dogstar Foundation [20], which regularly arranges mobile sterilization units. The findings on dog demographics show many similarities with previous studies on the dog population of Mirigama [22] and Colombo [47]. Male dogs were overrepresented in the owned dog populations of the three cities, representing also a bigger proportion of the vaccinated population in the Mirigama study. As in Negombo, this study also reported high proportions of owned dogs among the studied population, and lower proportions of young dogs (less than 1 year of age). The Colombo study also reported higher proportions of neutered females, compared to males. Conversely, while owned dogs in Negombo were seldom neutered, high percentages of neutered owned dogs were described in Colombo. In conclusion, very similar demographics could be found from the studies in the three cities, and although it may not be representative across the nation, the characterization of the dog population in each setting is a vital step for the development of successful vaccination campaigns.

As a further step, the regression model was able to identify several factors associated with a failure to attend a static point, notably the distance between household and the SP, and the young age of the dog. It is hoped that this information can be used to inform the roll out of future vaccination strategies in Sri Lanka. In a similar study using the same technique for a campaign in Malawi, Mazeri et al. [11] found that there was a decreasing likelihood of attending a SP with increasing distance from home to said SP, in dogs of young age, and in animals with poor health. These results parallel the findings from the Negombo study. Distance was also the sixth most common reason dog owners gave for not attending a SP, and the deterring effect of distance between domicile and healthcare facilities has been reported in previous studies [48–51]. The young age of the dog appears as the fifth most common quoted reason for not attending the SP. According to the regression analysis, odds of dogs under one year of age to be brought to a SP were 3.7 times lower compared to adult dogs. This finding has been observed in other studies where a misleading “too young to vaccinate” attitude prevails, leading to dogs remaining unimmunised in the population for several months [52]. The reduction in the odds of attendance with dogs with poor Body Condition Score (BCS) might be linked to owner care as a factor. In addition, the most common reasons the owners gave for not attending a SP were unawareness, unavailability and dog handling problems. These three reasons were also the most common answers to that same question in studies performed in different settings [11, 53–56]. However, answers conveying distrust in the campaign or the procedure, and/or lack of rabies disease knowledge, which were common in some of these studies, represented only a small percentage of the reasons provided by Negombian owners. This might be related to the high level of rabies awareness of the Sri Lankan population [18]. Increased focus into awareness prior to the vaccination and the arrangement of educational campaigns on responsible dog ownership and handling techniques could potentially allow more owners, willing to vaccinate their dogs, to attend SP campaigns. Increasing the number of SP deployed across the region might increase attendance rates by reducing distance to households. In addition, efforts to increase community awareness about the fact that puppy vaccination is a safe and necessary procedure are also required in order to effectively decrease the number of susceptible dogs in the population.

The 2016 vaccination campaign provided large amounts of data on the local dog population, enabling to investigate the local dog demographics. Moreover, the GPS coordinate recordings allowed the inclusion of additional variables of geospatial nature, with the goal of enriching the regression analysis and increasing its explanatory power. The unexplained variation reducing the predictability of the model could be due to the poor resolution of the geographic information data available for Sri Lanka, or due to the lack thereof, preventing the inclusion of other potentially explanatory variables, such as poverty data. Vaccination coverage results from 9 wards were unusable due to data collection unreliability, losing the ability to completely assess the breadth of the city-wide campaign. D2D data regarding the vaccination status of dogs shows similar percentages of successfully vaccinated dogs in these 9 wards (65%, CI 63.4–66.3) compared to the rest of the wards (72%, CI 69.2–75.3), and dog demographics were nearly indistinguishable between them. For these reasons, we expect the vaccination efforts to be successful and homogeneous throughout the working area, even though vaccination coverages could not be produced for these 9 wards due to inaccurate data. We acknowledge this fact as a limitation of our study, and suggest increasing overall staff competency appointing and training additional back-up supervisors for the different campaign stages. The vaccination coverages reported in this study might be underestimates, as collars and paint, used to mark vaccinated dogs, could have been lost or worn off respectively due to the timespan between stages. Lastly, any survey method chosen to assess vaccination coverage includes the possibility of missing some dogs in the population. In this study, this was minimized by the use of small vehicles and the ability to observe the urban layout through the use of the Mission Rabies App, identifying inspection routes dynamically to ensure thorough coverage and preventing path retracing.

## Conclusion

This report describes a high numbers, high coverage mass dog vaccination campaign in Negombo, Sri Lanka. Over 7800 dogs were vaccinated, achieving a coverage of 75.8%. The data collected during the campaign allowed to study the local dog population and identify factors influencing attendance to static vaccination points. This campaign serves as a working template that can be used to develop dog vaccination strategies in other parts of the country. In addition, the insights obtained on dog ecology and barriers of attendance can be used to increase the cost-efficiency of said campaigns, facilitate their development, and improve their coverage.

## Supplementary information

**Additional file 1: Figure S1.** Proportions of the dog population according to different sets of variables. Each 2 by 2 table describes the proportions of the dog population according to each pair of variables. The *P* value obtained for the test of independence between both variables is shown.

**Additional file 2: Figure S2.** Results from the univariable regression analysis. Variables whose P value was under 0.2 were considered for the model and included in the models created mostly by forward selection. The age category was divided into adult (over one year of age) and young (less than one year) for the final model due to low counts of puppy dogs.

## Data Availability

The vaccination and survey datasets generated and analysed during the current study are not publicly available. This data is held by Mission Rabies. GIS datasets are available from the respective sources as described in the “Materials and Methods: Data sources: Other sources” section.

## Abbreviations

AIC: Akaike information criterion
AUC: Area under the receiving operator curve
BCS: Body condition score
CI: Confidence Interval
D2D: Door-to-door
GPS: Global positioning system
PEP: Post-exposure prophylaxis
SP: Static point
WHO: World Health Organization

## References

[1] Hampson K, Coudeville L, Lembo T, Sambo M, Kieffer A, Attlan M, Barrat J, Blanton J, Briggs D, Cleaveland S, Costa P, Freuling C, Hiby E, Knopf L, Leanes F, Meslin F, Metlin A, Miranda M, Müller T, Nel L, Recuenco S, Rupprecht C, Schumacher C, Taylor L, Vigilato M, Zinsstag J, Dushoff J (2015). Estimating the global burden of endemic canine rabies. PLOS Negl Trop Dis.

[2] World Health Organization, Rabies fact sheet. (2017). http://www.who.int/mediacentre/factsheets/fs099/en. .

[3] Hemachudha T, Ugolini G, Wacharapluesadee S, Sungkarat W, Shuangshoti S, Laothamatas J (2013). Human rabies: neuropathogenesis, diagnosis, and management. Lancet Neurol.

[4] Fooks A, Banyard A, Horton D, Johnson N, McElhinney L, Jackson A (2014). Current status of rabies and prospects for elimination. Lancet.

[5] Hampson K, Dushoff J, Cleaveland S, Haydon D, Kaare M, Packer C, Dobson A (2009). Transmission dynamics and prospects for the elimination of canine rabies. PLoS Biol.

[6] Cleaveland S, Kaare M, Knobel D, Laurenson MK (2006). Canine vaccination – providing broader benefits for disease control. Vet Microbiol.

[7] Lembo T, Hampson K, Kaare M, Ernest E, Knobel D, Kazwala R, Haydon D, Cleaveland S (2010). The feasibility of canine rabies elimination in Africa: dispelling doubts with Data. PLoS Negl Trop Dis.

[8] Morters M, McKinley T, Horton D, Cleaveland S, Schoeman J, Restif O, Whay H, Goddard A, Fooks A, Damriyasa I, Wood J (2014). Achieving population-level immunity to rabies in free-roaming dogs in Africa and Asia. PLoS Negl Trop Dis.

[9] WHO Expert Consultation on Rabies: second report (2013). http://apps.who.int/iris/handle/10665/85346. Accessed 16 Nov 2018. 24069724

[10] Coleman P, Dye C (1996). Immunization coverage required to prevent outbreaks of dog rabies. Vaccine.

[11] Mazeri S, Gibson A, Meunier N, Bronsvoort B, Handel I, Mellanby R, Gamble L (2018). Barriers of attendance to dog rabies static point vaccination clinics in Blantyre, Malawi. PLoS Negl Trop Dis.

[12] Tenzin T, Ahmed R, Debnath N, Ahmed G, Yamage M (2015). Free-roaming dog population estimation and status of the dog population management and rabies control program in Dhaka City, Bangladesh. PLoS Negl Trop Dis.

[13] Gibson A, Ohal P, Shervell K, Handel I, Bronsvoort B, Mellanby R, Gamble L. Vaccinate-assess-move method of mass canine rabies vaccination utilising mobile technology data collection in Ranchi, India. BMC Infect Dis. 2015;15(1). 10.1186/s12879-015-1320-2 PMC4696259 26715371

[14] Léchenne M, Oussiguere A, Naissengar K, Mindekem R, Mosimann L, Rives G, Hattendorf J, Moto D, Alfaroukh I, Zinsstag J (2016). Operational performance and analysis of two rabies vaccination campaigns in N’Djamena, Chad. Vaccine.

[15] Gibson A, Handel I, Shervell K, Roux T, Mayer D, Muyila S, Maruwo G, Nkhulungo E, Foster R, Chikungwa P, Chimera B, Bronsvoort B, Mellanby R, Gamble L (2016). The vaccination of 35,000 dogs in 20 working days using combined static point and door-to-door methods in Blantyre, Malawi. PLoS Negl Trop Dis.

[16] Health Statistical Bulletin. (2015). http://www.health.gov.lk/moh_final/english/public/elfinder/files/publications/AHB/2017/AHB%202015.pdf. Accessed 16 Nov 2018.

[17] Abela-Ridder B, Lionel Harischandra P, Gunesekera A, Janakan N, Gongal G (2016). Sri Lanka takes action towards a target of zero rabies death by 2020. WHO South-East Asia. J Public Health.

[18] Kumarapeli V, Awerbuch-Friedlander T (2009). Human rabies focusing on dog ecology — a challenge to public health in Sri Lanka. Acta Trop.

[19] Mission Rabies Sri Lanka vaccination campaigns. http://www.missionrabies.com/projects/sri-lanka (2017). Accessed 16 Nov 2018.

[20] Dogstar Foundation. https://www.dogstarfoundation.com (2018). Accessed 16 Nov 2018.

[21] Census of Population and Housing of Sri Lanka (2012). http://www.statistics.gov.lk/PopHouSat/CPH2011/Pages/Activities/Reports/District/Gampaha/A4.pdf. .

[22] Matter H, Wandeler A, Neuenschwander B, Harischandra L, Meslin F (2000). Study of the dog population and the rabies control activities in the Mirigama area of Sri Lanka. Acta Trop.

[23] Gibson A, Mazeri S, Lohr F, Mayer D, Bailey J, Wallace R, Handel I, Shervell K, Bronsvoort B, Mellanby R, Gamble L (2018). One million dog vaccinations recorded on mHealth innovation used to direct teams in numerous rabies control campaigns. PLOS ONE.

[24] World Weather Online. https://worldweatheronline.com (2016). Accessed 16 Nov 2018.

[25] WorldPop. www.worldpop.org.uk (2015). Accessed 16 Nov 2018.

[26] Socioeconomic Data and Applications Center. http://sedac.ciesin.columbia.edu (2018). Accessed 16 Nov 2018.

[27] World Bank Group. https://datacatalog.worldbank.org/ (2012). Accessed 16 Nov 2018.

[28] Kahle D, Wickham H (2013). Ggmap: spatial visualization with ggplot2. R Journal.

[29] Open Street Map. https://openstreetmap.org (2018). Accessed 16 Nov 2018.

[30] R Core Team (2017). R: a language and environment for statistical computing. R Foundation for Statistical Computing, Vienna, Austria. https://www.R-project.org.

[31] Ordoñez C, Martínez J, Rodríguez-Pérez J, Reyes A (2011). Detection of outliers in GPS measurements by using functional-Data analysis. J Surv Eng.

[32] Hennig, C. (2018). Fpc: flexible procedures for clustering. R package version 2.1–11. https://CRAN.R-project.org/package=fpc

[33] Bivand, R. and Rundel, C. (2017). Rgeos: Interface to geometry engine - open source ('GEOS'). R package version 0.3–26. https://CRAN.R-project.org/package=rgeos

[34] Bivand, R., Keitt, T., and Rowlingson, B. (2018). Rgdal: bindings for the 'Geospatial' Data abstraction library. R package version 1.2–18. https://CRAN.R-project.org/package=rgdal

[35] Chapman D (1954). The estimation of biological populations. Ann Math Stat.

[36] Tyers, M. (2016). Recapr: estimating, testing, and simulating abundance in a mark-recapture. R package version 0.3.9002. https://rdrr.io/github/mbtyers/recapr/

[37] Sunil Arya, David Mount, Samuel E. Kemp and Gregory Jefferis (2018). RANN: fast nearest neighbour search (wraps ANN library) using L2 metric. R package version 2.6. https://CRAN.R-project.org/package=RANN

[38] Robert J. Hijmans (2017). Raster: geographic Data analysis and modeling. R package version 2.6–7. https://CRAN.R-project.org/package=raster

[39] Box G, Tidwell P (1962). Transformation of the independent variables. Technometrics.

[40] National Meteorological Library and Archive Fact sheet 3. – Water in the atmosphere (2012). https://www.metoffice.gov.uk/binaries/content/assets/mohippo/pdf/f/c/fact_sheet_no._3.pdf. Accessed 16 Nov 2018.

[41] Mount J, Zumel N. (2018). Vtreat: a statistically sound 'data.frame' processor/conditioner. R package version 1.3.0. https://CRAN.R-project.org/package=vtreat

[42] Max Kuhn. Contributions from Jed Wing, Steve Weston, Andre Williams, Chris Keefer, Allan Engelhardt, Tony Cooper, Zachary Mayer, Brenton Kenkel, the R Core Team, Michael Benesty, Reynald Lescarbeau, Andrew Ziem, Luca Scrucca, Yuan Tang, Can Candan and Tyler Hunt (2018). Caret: classification and regression training. R package version 6.0–80. https://CRAN.R-project.org/package=caret

[43] Stone M (1974). Cross-Validatory choice and assessment of statistical predictions. J R Stat Soc Ser B Methodol.

[44] Akaike H (1974). A new look at the statistical model identification. IEEE Trans Autom Control.

[45] Sing T, Sander O, Beerenwinkel N, Lengauer T (2005). ROCR: visualizing classifier performance in R. Bioinformatics.

[46] Knobel D, Cleaveland S, Coleman P, Fevre E, Meltzer M, Miranda M, Shaw A, Zinsstag J, Meslin F (2005). Re-evaluating the burden of rabies in Africa and Asia. Bull World Health Organ.

[47] Seneviratne M, Subasinghe D, Watson P (2016). A survey of pet feeding practices of dog owners visiting a veterinary practice in Colombo, Sri Lanka. Vet Med Sci.

[48] Stock R (1983). Distance and the utilization of health facilities in rural Nigeria. Soc Sci Med.

[49] Müller I, Smith T, Mellor S, Rare L, Genton B (1998). The effect of distance from home on attendance at a small rural health Centre in Papua New Guinea. Int J Epidemiol.

[50] Buor D (2003). Analysing the primacy of distance in the utilization of health services in the Ahafo-Ano south district, Ghana. Int J Health Plann Manag.

[51] Kumar S, Dansereau E, Murray C (2014). Does distance matter for institutional delivery in rural India?. Appl Econ.

[52] Morters M, McNabb S, Horton D, Fooks A, Schoeman J, Whay H, Wood J, Cleaveland S (2015). Effective vaccination against rabies in puppies in rabies endemic regions. Vet Rec.

[53] Muthiani Y, Traoré A, Mauti S, Zinsstag J, Hattendoorf J (2014). Low coverage of central point vaccination against dog rabies in Bamako, Mali. Prev Vet Med.

[54] Bardosh K, Sambo M, Sikana L, Hampson K, Welburn S (2014). Eliminating rabies in Tanzania? Local understandings and responses to mass dog vaccination in Kilombero and Ulanga districts. PLoS Negl Trop Dis.

[55] Castillo-Neyra R, Brown J, Borrini K, Arevalo C, Levy M, Buttenheim A, Hunter G, Becerra V, Behrman J, Paz-Soldan V (2017). Barriers to dog rabies vaccination during an urban rabies outbreak: qualitative findings from Arequipa, Peru. PLoS Negl Trop Dis.

[56] Mulipukwa C, Mudenda B, Mbewe A (2017). Insights and efforts to control rabies in Zambia: evaluation of determinants and barriers to dog vaccination in Nyimba district. PLoS Negl Trop Dis.

