# Supplementary figures and images for: Development of a high number, high coverage dog rabies vaccination programme in Sri Lanka
Source: BMC Infect Dis. 2019 Nov 20;19:977. doi: 10.1186/s12879-019-4585-z (PMC6868729; doi:10.1186/s12879-019-4585-z)

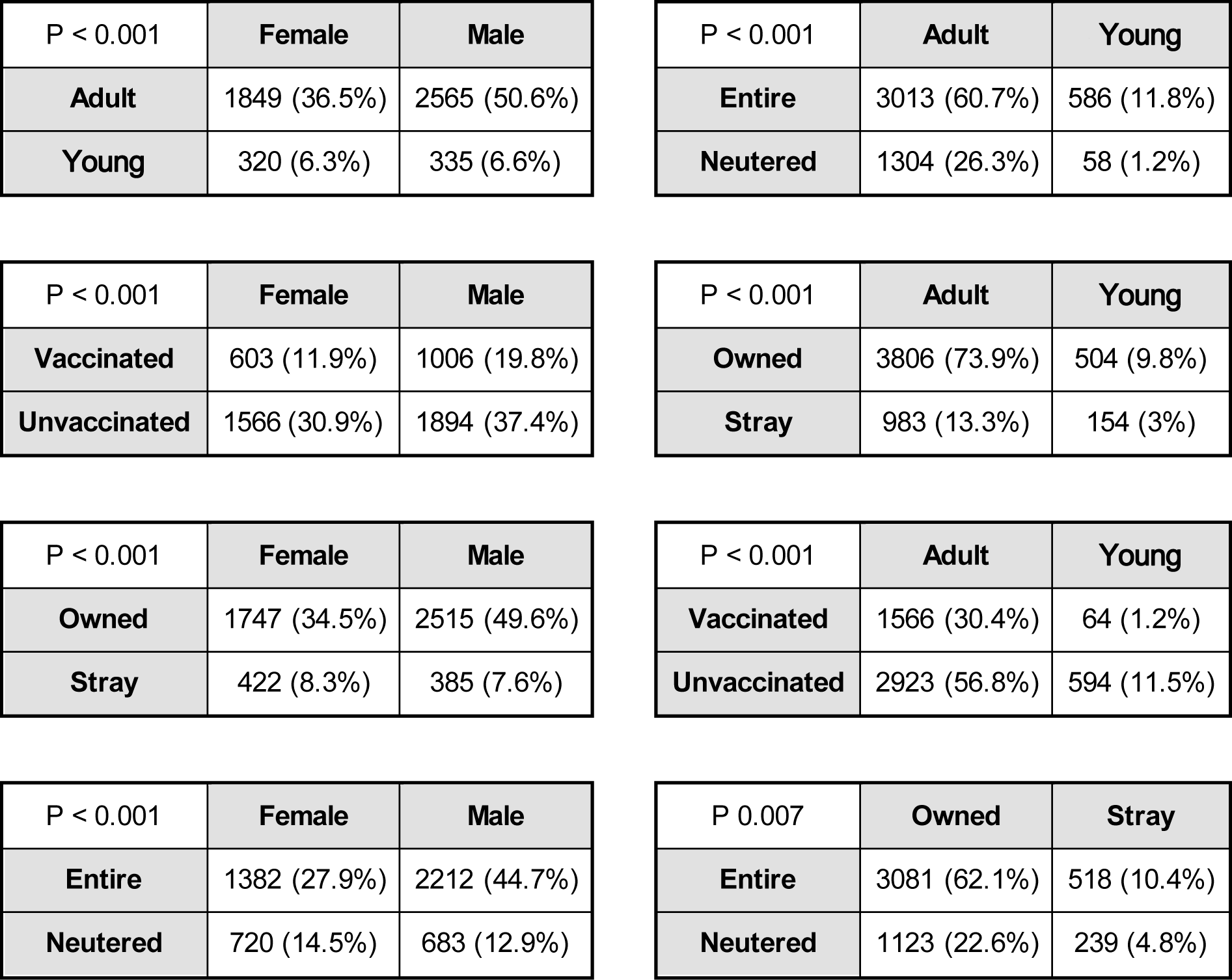

Supplement: Supplementary file 1 — Additional file 1: Figure S1. Proportions of the dog population according to different sets of variables. Each 2 by 2 table describes the proportions of the dog population according to each pair of variables. The P value obtained for the test of independence between both variables is shown. [file 12879_2019_4585_MOESM1_ESM.tif]
